# Highly Elevated Serum Hepcidin in Patients with Acute Myeloid Leukemia prior to and after Allogeneic Hematopoietic Cell Transplantation: Does This Protect from Excessive Parenchymal Iron Loading?

**DOI:** 10.1155/2011/491058

**Published:** 2011-05-05

**Authors:** Ann-Kathrin Eisfeld, Mark Westerman, Rainer Krahl, Sabine Leiblein, Uwe Gerd Liebert, Marianne Hehme, Daniel Teupser, Dietger Niederwieser, Haifa Kathrin Al-Ali

**Affiliations:** ^1^Department of Hematology/Oncology, University of Leipzig, Johannesallee 32a, 04103 Leipzig, Germany; ^2^Intrinsic LifeSciences LLC, La Jolla, CA 92037, USA; ^3^Institute of Virology, University of Leipzig, 04103 Leipzig, Germany; ^4^Novartis Pharma GmbH, 90429 Nuernberg, Germany; ^5^Institute of Laboratory Medicine, University of Leipzig, 04103 Leipzig, Germany

## Abstract

Hepcidin is upregulated by inflammation and iron. Inherited (HFE genotype) and treatment-related factors (blood units (BU), Iron overload) affecting hepcidin (measured by C-ELISA) were studied in 42 consecutive patients with AML prior to and after allogeneic hematopoietic cell transplantation (HCT). *Results*. Elevated serum ferritin pre- and post-HCT was present in all patients. Median hepcidin pre- and post-HCT of 358 and 398 ng/mL, respectively, were elevated compared to controls (median 52 ng/mL) (*P* < .0001). Liver and renal function, prior chemotherapies, and conditioning had no impact on hepcidin. Despite higher total BU after HCT compared to pretransplantation (*P* < .0005), pre- and posttransplant ferritin and hepcidin were similar. BU influenced ferritin (*P* = .001) and hepcidin (*P* = .001). No correlation of pre- or posttransplant hepcidin with pretransplant ferritin was found. HFE genotype did not influence hepcidin. *Conclusions*. Hepcidin is elevated in AML patients pre- and post-HCT due to transfusional iron-loading suggesting that hepcidin synthesis remains intact despite chemotherapy and HCT.

## 1. Introduction

Hepcidin, a 25-amino acid antimicrobial peptide synthesized in the liver, is the central iron-regulatory hormone that mediates the homeostasis of extracellular iron concentrations [1, 2]. Studies have confirmed its role as the negative regulator of iron absorption, recycling, and release from stores. Hepcidin regulates iron export into plasma from tissues involved in iron storage or transport such as duodenal enterocytes, hepatocytes, macrophages, and the syncytial trophoblasts of the placenta [3]. Hepcidin binds to the iron exporter ferroportin and induces its internalization and degradation. Systemic iron requirements and infectious as well as inflammatory stimuli modulate hepcidin expression [4, 5]. Its transcription is upregulated by cytokines such as interleukin-6 and interleukin-1 as well as by iron and is downregulated by iron deficiency, ineffective erythropoiesis, and hypoxia [4, 6]. Because of its sensitivity to inflammatory stimuli and its effect on iron export from various tissues, hepcidin plays an essential role for iron disorders such as anemia of inflammation [7]. Hepcidin expression is also associated with transfusional iron overload in *β*-thalassemia and other iron-loading anemias [8].

Additionally, evidence indicates that the hemochromatosis gene HFE might be required for the synthesis of hepcidin. Recent studies with animal models of hemochromatosis suggest an important role of HFE protein as an iron sensor and upstream regulator of hepcidin in hepatocytes. Hepatocyte HFE is necessary for signaling to hepcidin, presumably as a constituent of a larger iron-sensing complex [9–12].

Iron overload, mainly due to multiple blood transfusions, is a common complication in recipients of hematopoietic cell transplantation (HCT) [13]. Iron overload increases the risk of infections, veno-occlusive disease, and hepatic dysfunction after HCT. An increase of pretransplant serum ferritin levels, which usually reflects body iron stores, has been shown to lower disease-free survival following HCT and might increase the risk of graft-versus-host disease (GvHD). However, additional studies are needed to determine the influence of iron overload on long-term morbidity and mortality in allogeneic HCT survivors [14].

In patients with acute myeloid leukemia (AML) receiving HCT, both inherited and treatment-related factors might influence hepcidin expression. Whether hepcidin synthesis and regulation remain intact despite intensive AML-chemotherapy and HCT is yet unknown. Therefore, the impact of pretransplant body iron load, blood transfusions, HFE genotype as well as chemotherapy, conditioning regimen, and graft-versus-host disease (GVHD) on serum hepcidin levels was analyzed in patients with AML prior to and after allogeneic HCT.

## 2. Materials and Methods

### 2.1. Study Design

Between February 2008 and February 2009, 42 consecutive patients with AML in complete remission who received allogeneic HCT at the University of Leipzig, Germany, and survived three months were included. All patients were assessed clinically and biochemically on two occasions: ten days prior to and at a median of 3 (range 3–5) months after allogeneic HCT. All medications including antibiotics, antifungal, and immunosuppressive drugs were recorded. The study was conducted in accordance with the Declaration of Helsinki [15]. Patients gave written informed consent.

### 2.2. Patients

Median age was 57 (range 18–70) years. 23 (54.8%) were male, and 19 (45.2%) patients were female. Median number of AML-chemotherapies prior to HCT was 3 (range, 0–5). Conventional conditioning with 12 Gray total body irradiation (TBI) and cyclophosphamide 120 mg/kg was given to 13 (31%) patients while 29 (69%) of patients received HCT following reduced intensity conditioning (RIC) with fludarabin 30 mg/m^2^/day for 3 days and 2 Gray TBI applied once followed by immunosuppression with cyclosporine (through level >200 ng/mL) and mycophenolate mofetil. Donors were matched related (MRD) in 8 (19%) and matched unrelated (MUD) in 34 (81%) patients. Acute GVHD confirmed by biopsy of at least one involved site was graded according to the Glucksberg-Seattle criteria [16]. 14 (33.3%) patients developed grade > II acute GVHD of the skin while 7 (16.7%) patients suffered grade > II acute GVHD of the liver.

Patients received a median of 22 (range 8–95) units of blood pre-HCT and a median total of 30 (14–120) units after HCT.  Table 1 summarises patient characteristics.

### 2.3. Body Iron

For assessment of body iron stores, serum ferritin was measured. At the time of assessment, patients were in a stable clinical condition without signs of infection and a concomitant C-reactive protein (CRP) <10 mg/L. Normal reference value for ferritin was 30–400 ng/mL.

### 2.4. Hepcidin

Concomitant with the measurement of serum ferritin, serum hepcidin was measured by a hepcidin competitive enzyme-linked immunoassay (C-ELISA) at Intrinsic LifeSciences LLC, La Jolla, CA, USA, as previously described [17]. In healthy volunteers, the 5% to 95% range of hepcidin concentrations was 29–254 ng/mL for males (*n* = 65) and 17–286 ng/mL for females (*n* = 49) with median concentrations of 112 and 65 ng/mL, respectively (*P* < .001) [17].

In this study, serum hepcidin concentrations of 21 healthy volunteers matched for age and gender (6 male, 15 female, median age 57 years) were used as controls.

### 2.5. HFE Gene Analysis

Patients were screened for their HFE genotype prior to and after HCT. Mutations in DNA extracted from peripheral blood were detected by polymerase chain reaction (PCR) using hybridization probes and melting curve analysis as described previously [18].

### 2.6. Donor Cell Chimerism

After conventional conditioning, donor chimerism was analysed in unsorted bone marrow cells day 28 after HCT. For all patients who received RIC HCT, additionally flow-sorted T (CD3+)-, and CD34+-bone marrow cells at days 28, 56, 84, and at 3 months interval thereafter was monitored by fluorescence in situ hybridisation (FISH) for the XY chromosome in gender mismatched or PCR-based analysis of polymorphic microsatellite regions in gender-matched HCT. All patients expressed a full donor chimerism after HCT.

### 2.7. Liver and Renal Function

Alanine aminotransferase (ALT), aspartate aminotransferase (AST), alkaline phosphates (AP), and bilirubin were evaluated regularly. Normal laboratory reference value for ALT/AST, AP, and bilirubin were ≤0.6 mmoL/L, <1.74 *μ*kat/L, and <17 *μ*moL/L, respectively. Creatinine was used to monitor renal function. Normal reference value for creatinine was <104 *μ*moL/L (Table 1).

### 2.8. Statistical Analysis

For statistical analysis Wilcoxon signed-rank test and Mann-Whitney *U* test were used. Cox-Regression was used for multivariate analysis. For nonparametric correlations, we used the Spearman-Rho-Test and the Pearson-correlation. Data were analyzed using SPSS15 software. A *P*  value of <.05 was considered statistically significant.

## 3. Results

### 3.1. Body Iron Stores prior to and after HCT

Prior to allogeneic HCT, excess body iron was present in all patients with a median serum ferritin of 1945 (range 617–6981) ng/mL. Patients received a median of 22 (range 8–95) units of blood. After HCT, the median serum ferritin of 2260 (range 807–7595) ng/mL was not statistically higher than the pre-transplant level although the number of blood transfusions after HCT reached a median of 30 (range 14–120) units which was significantly more than that received prior to HCT (*P* < .0005). The number of blood transfusions prior to HCT strongly correlated with the pretransplant serum ferritin (*P* = .001). Again, the posttransplant serum ferritin was strongly dependent on the cumulative number of blood units transfused (*P* < .001).

### 3.2. HFE Genotypes prior to and after HCT

Data on HFE gene status were available in 41 patients prior to and in 40 patients after HCT. In the pre-transplantation setting, mutations were found in 19 (46.0%) patients. The most frequently occurring HFE mutation was heterozygosity (het) for H63D (*n* = 11), followed by het C282Y (*n* = 3), and het S65C (*n* = 1). Homozygosity (homo) for H63D was detected in 4 patients. After HCT, HFE mutations were detected in 15 (37.5%) patients. Again heterozygosity for H63D occurred most frequently (*n* = 12), followed by het S65C (*n* = 2), and het C282Y (*n* = 1). Compound-het was detected in one patient after allogeneic HCT. Interestingly, in 18 patients the HFE genotype changed after HCT (Table 2). HFE genotype at any time point had no influence on serum ferritin.

### 3.3. Hemoglobin (Hb) prior to and after HCT

Prior to HCT, Hb with a median of 10.3 (range 6.8–14.5) g/dL was similar to Hb levels after HCT (median 10.1 (range 6.7–13.4) g/dL). There was a negative correlation between Hb levels and serum ferritin prior to HCT (*P* = .002). Similarly, higher ferritin values were associated with lower Hb levels after transplantation (*P* = .01).

### 3.4. Hepcidin Levels prior to and after HCT

The median hepcidin concentration of 52.1 (range 8.3–130.8) ng/mL in the 21 healthy volunteers in this analysis was comparable to the published values in healthy volunteers measured with this assay [17]. 

**Figure 1:**
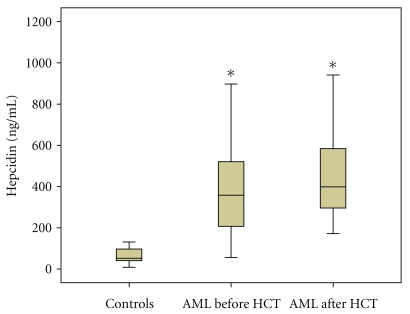
Hepcidin concentrations in controls and in AML patients before and after allogeneic HCT (*n* = 42); *P < .0001, each versus controls.

Patients with AML demonstrated highly elevated serum hepcidin levels both in the pre-transplant (median 358 (range 56–1096) ng/mL) and post-transplant setting (median 398 (range 172–941) ng/mL) compared to the control group (*P* < .0001). There was no statistically significant difference between median hepcidin levels prior to and after HCT (*P* = .4) (Figure 1).

#### 3.4.1. Correlation of Hepcidin Levels with Blood Transfusions

**Figure 2:**
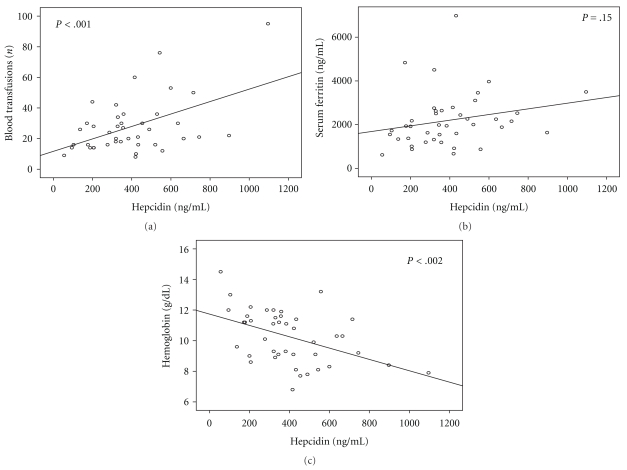
Correlation of serum hepcidin levels with number of blood transfusions (a), serum ferritin values (b), and hemoglobin levels (c) before HCT.

Hepcidin levels before HCT strongly correlated with the number of blood transfusions prior to HCT (*P* = .001) (Figure 2(a)). Similarly, post-transplant hepcidin values significantly correlated with both the number of blood units transfused prior to transplantation (*P* = .008) and the total units of blood transfused (*P* < .001).

#### 3.4.2. Correlation of Hepcidin Levels with Body Iron

Surprisingly in the pre-transplant setting, and although the number of blood units transfused strongly correlated with the serum ferritin, no statistical correlation was found between serum ferritin values prior to HCT and serum hepcidin concentrations prior to or after transplantation (Figure 2(b)).

#### 3.4.3. Correlation of Serum Hepcidin with Hb Levels

Hepcidin concentrations significantly correlated inversely with Hb values (*P* = .002) (Figure 2(c)). A similar association could be found between hepcidin and Hb values after HCT (*P* = .001).

#### 3.4.4. Effect of HFE Genotype on Hepcidin Levels

In our cohort, HFE genotype prior to or after HCT had no impact on hepcidin values at any time point.

#### 3.4.5. Effect of Other Patient Characteristics on Hepcidin Levels

In univariate analysis, age and gender had no significant influence on hepcidin values before or after HCT. Similarly, liver and renal functions, number of prior chemotherapies, and type of conditioning regimen had no impact on hepcidin levels either before or after HCT.

### 3.5. Outcome after HCT

After a median followup of 21 (range 17–28) months, the probability of survival and leukemia-free survival (LFS) at 2 years were 66% and 64%, respectively. The incidences of relapse and nonrelapse mortality (NRM) at 2 years were 20%. In this analysis, pre- and post-HCT serum ferritin and hepcidin levels as well as the number of blood transfusions did not correlate with survival, LFS, relapse, or NRM. Acute graft-versus-host disease (GVHD) which occurred in 35 (83%) patients was limited to acute cutaneous GVHD stage I and II in 31 patients. Chronic GVHD (limited, *n* = 4, extensive, *n* = 18) was present in 22 (53%) patients. Again, there was no association between the occurrence and the severity of GVHD on one side and pre- or post-HCT body iron or hepcidin levels on the other side. In the first 100 days post-HCT, 12 (29%) patients suffered a nonlethal grade 3 infection according to common toxicity criteria. In the entire cohort, serum ferritin or hepcidin values did not influence the occurrence of severe infections.

## 4. Discussion

Evidence for iron overload as determined by serum ferritin concentrations was found in all polytransfused AML patients. Serum ferritin is a practical measure of body iron that can easily be applied and is supposed to represent a reliable marker of body iron stores [19–21]. Although factors such as infection, acute and chronic inflammation, and alcohol abuse can exert an influence on individual values, both the stable clinical condition and the concomitant low CRP in all patients suggest that the serum ferritin mainly reflected body iron stores.

It is not surprising that serum ferritin at all time points strongly correlated with the number of blood transfusions as iron overload, commonly observed in recipients of allogeneic HCT, is mainly attributed to multiple blood transfusions. But other factors as overexpression of the growth and differentiation factor (GDF-15) which inhibits the production of hepcidin in the liver have been discussed as possible contributors to excess body iron in thalassemia [22].

In this study, patients with AML compared to healthy volunteers demonstrated highly elevated serum hepcidin levels strongly correlating with the number of blood transfusions both in the pre- and post-transplant setting. This finding suggests that transfusional iron remains a dominant regulator of hepcidin in these patients and that hepcidin synthesis and regulation remain intact in polytransfused patients with AML undergoing allogeneic HCT irrespective of the number of prior chemotherapies, type of conditioning regimen, or antifungal treatments.

Usually, hepcidin and serum ferritin respond similarly to inflammation and changes in iron stores, and this is reflected in the strong correlation between hepcidin and ferritin in healthy volunteers with hepcidin responses taking place on the time scale of a few hours, whereas changes in ferritin concentrations are much slower [23]. However, no correlation of pre- or post-HCT hepcidin levels with serum ferritin values prior to HCT was found despite the fact that both serum ferritin and hepcidin were strongly affected by the number of blood units transfused. Also, pre- and post-transplant serum ferritin and hepcidin values were statistically similar despite significantly higher cumulative blood units received after HCT. These data suggest that hepcidin represents a compensatory mechanism that counteracts transfusional iron input. Hepcidin is known to bind to ferroportin, leading to intracellular retention of iron in macrophages and to a reduction of extracellular iron bound to transferrin or serum ferritin [24]. This raises the fascinating question of whether elevated hepcidin values in the presence of transfusional iron loading might protect from excessive parenchymal iron overload and subsequent organ damage.

An elevated hepcidin expression as a proposed cause of anemia of chronic inflammation [7] might explain the inverse correlation of hepcidin levels with Hb values observed in this study and highlight the complexity of hepcidin regulation.

As HFE gene mutations have a high frequency in population of European descent [9–11], patients in this study were screened prior to and after HCT for HFE gene mutations to determine whether HFE genotype contributed to excess iron and to assess the impact of HFE gene mutations on hepcidin concentrations. As all patients after HCT expressed full donor chimerism, the HFE genotype after HCT must be considered to reflect donor genotype. HFE deletions or mutations have been associated with a reduction of liver hepcidin expression [12, 25–27]. However, it is presently controversial whether HFE is indispensable for hepcidin activation in response to inflammatory stimuli [28–30]. Mutations of HFE account for most but not all cases of hemochromatosis [31]. The most common mutation, C282Y, is associated with disruption of a disulfide bond in HFE that is critical for its binding to *β*_2_ microglobulin [32]. This interaction is required for the stabilisation, transport, and expression of HFE on the cell surface and endosomal membranes where HFE interacts with the transferrin receptor 1 (TfR1). The H63D mutation, a common HFE mutation whose pathogenic significance is still uncertain, does not impair interaction between HFE and TfR1. As the H63D HFE mutation was the only mutation commonly detected in our cohort, this might in part explain why HFE genotype had no influence on serum hepcidin levels.

Similarly, the missing association between HFE genotype and serum ferritin in our cohort is in accordance with published data. Veneri et al. studied the prevalence of 12 mutations of the HFE gene and its correlation with the iron status in 82 adult patients with acute leukemia of whom 58.5% were affected by acute myeloid leukemia (AML) [33]. 32.9% of the patients had at least one HFE gene mutation and mean serum ferritin levels were increased at diagnosis. However, there was no difference between patients positive or negative for the HFE mutations in terms of serum ferritin levels. The study therefore highlighted the presence of iron overload in many AML patients but did not support the evidence of an association between HFE mutations and iron overload in acute leukemia [33]. 

Increased pre-transplant iron overload has been demonstrated to represent negative prognostic factors in patients with myelodysplastic syndromes or secondary AML [34], thalassemia, and other hematologic disorders such as acute leukemias [35, 36] receiving HCT irrespective of the type of conditioning [35–37].

The impact of an elevated hepcidin level on outcome after HCT is not yet known. But few data suggest that pre-transplant serum hepcidin levels might predict the risk of early infectious bacterial complications after allogeneic HCT [38]. 

Although the aim of this work was not to study the impact of hepcidin values on outcome after HCT, nevertheless no negative impact of an elevated pre-transplant hepcidin concentration on outcome, the incidence or severity of acute or chronic GVHD, or early infectious complications after HCT could be detected in this cohort. It is important to keep in mind that this was a small series of patients surviving the first three months after HCT. Nevertheless, this observation merits further investigation with a higher number of patients.

## 5. Conclusions

For the first time, we describe highly elevated serum hepcidin concentrations in AML patients with transfusional iron load both prior to and after HCT using the C-ELISA for human serum hepcidin assessment. This suggests that hepcidin synthesis and upregulation remain intact despite intensive chemotherapy and HCT. The weak correlation between pre-transplant serum ferritin and both pre- or post-transplant serum hepcidin is highly interesting although both pre- and post-transplant levels of serum ferritin and hepcidin were similar and correlated, as would be expected, with the number of blood units received. So Overexpression of hepcidin might represent a compensatory mechanism counteracting transfusional iron input.

Actually, overexpression of hepcidin might play an important protective role in this setting as it might prevent an increased ferroportin-mediated iron export from macrophages thereby reducing the severity of parenchymal iron loading and damage. In contrast to ferritin, a marker of iron stores, changes in hepcidin concentrations are frequently the cause of iron disorders. Thus, measurements of hepcidin concentrations in addition to serum ferritin might be highly informative in understanding iron homeostasis in the setting of HCT. To establish the exact impact of serum hepcidin on outcome after HCT, large prospective studies are warranted.

## Figures and Tables

**Table 1:** Patient characteristics at baseline (*n* = 42).

Parameters	Results
Mean age, years (range)	57 (18–70)

Gender, *n* (%)	
Male	23 (54.8)
Female	19 (45.2)

Donor, *n* (%)	
MRD	8 (19%)
MUD	34 (81%)

Conditioning, *n* (%)	
Conventional	13 (31.0)
RIC	29 (69.0)

Acute GVHD, *n* (%)	
Skin	
No	7 (16.7)
Yes	35 (83.3)
Grade I	21
Grade II	10
Grade III	4
Liver	
No	35 (83.3)
Yes	7 (16.7)
Grade I	6
Grade II	1
Grade III	0

Median number of blood transfusions (range)	
Prior to HCT	22 (8– 95)
After HCT	30 (14–120)

Median serum ferritin (range) ng/mL	
Prior to HCT	1945 (617–6981)
After HCT	2260 (807–7595)

Median serum hepcidin (range) ng/mL	
Prior to HCT	358 (56–1096 )
After HCT	398 (172–941)

MRD: matched related donor; MUD: matched unrelated donor; RIC: reduced intensity conditioning; GVHD: graft versus host disease; HCT: hematopoietic stem cell transplantation.

**Table 2:** HFE mutations before and after allogeneic HCT.

Patient ID	HFE mutations before HCT	HFE mutations after HCT
1	heth63D	wt
2	homoH63D	wt
3	wt	wt
4	wt	wt
5	wt	heth63D
6	wt	wt
7	wt	wt
8	wt	wt
9	wt	heth63D
10	wt	wt
11	hetC282Y	wt
12	wt	wt
13	heth63D	−1
14	wt	wt
15	hetC282Y	wt
16	wt	wt
17	wt	wt
18	hetH63D	hetH63D
19	wt	wt
20	hetH63D	wt
21	hetC282Y	wt
22	hetH63D	hetH63D
23	wt	hetH63D
24	hetH63D	hetH63D
25	hetH63D	hetH63D
26	hetH63D	hetH63D
27	wt	wt
28	wt	wt
29	hetH63D	hetS65C
30	homoH63D	wt
31	homoH63D	wt
32	hetS65C	wt
33	wt	hetH63D
34	wt	hetH63D
35	homoH63D	wt
36	hetH63D	hetS65C
37	hetH63D	hetH63D
38	wt	comp. het
39	wt	wt
40	wt	wt
41	wt	hetH63D
42	−1	−1

HFE: hereditary hemochromatosis gene; HCT: hematopoietic cell transplantation; het: heterozygous; homo: homozygous; comp. het: compound heterozygous; wt: wildtype.
